# Strengthening Tobacco-Free Educational Institutions Through School-Based Health Education: Implementation of Project Udaan in Vadodara, India

**DOI:** 10.7759/cureus.106997

**Published:** 2026-04-13

**Authors:** Hariprakash Hadial, Krupali Shah, Apurva Ratnu, Sreekala P. R., Imran A Malek

**Affiliations:** 1 Epidemiology and Public Health, Niramay Charitable Trust, Gandhinagar, IND; 2 Public Health, Fulcrum-Capitalising CSR, Vadodara, IND; 3 Public Health, Niramay Charitable Trust, Gandhinagar, IND

**Keywords:** adolescents, cancer prevention, community health research, head and neck cancer, health education, india, school health, smoking tobacco, tobacco-free schools, tobacco prevention

## Abstract

Background

Tobacco use remains a leading preventable cause of disease in India, with initiation commonly occurring during adolescence. Schools offer an effective platform for early prevention through structured health education. Project Udaan was developed as a school-based initiative to strengthen tobacco prevention awareness and support implementation of national tobacco control policies. The primary objective of this study was to describe the implementation of Project Udaan and assess changes in students’ knowledge related to tobacco use and oral cancer prevention among students in Grades 6 to 10 using a pre-test and post-test design.

Methods

Project Udaan was implemented across 100 urban and rural schools in Vadodara district, Gujarat, India, among students from Grades 6 to 10. The intervention included presentations, demonstrations, interactive activities, and awareness sessions conducted by trained educators. A pre-test and post-test design was used to assess knowledge improvement. Pre-test responses were obtained from 6,017 students and post-test responses from 6,047 students. Data were analyzed using descriptive statistics.

Results

A comparative analysis of pre- and post-test responses demonstrated substantial improvement in students’ knowledge across tobacco use and oral cancer prevention domains. Awareness of tobacco products increased from 5172/6017 (86.0%) to 5885/6047 (97.3%), and knowledge of the tobacco-oral cancer association improved from 5295/5963 (88.8%) to 5903/6047 (97.6%). Recognition of early oral cancer symptoms rose from 4038/5983 (67.5%) to 5643/6047 (93.3%), while understanding of peer pressure as a risk factor showed the greatest increase, from 2710/5926 (45.7%) to 5377/6047 (88.9%). Awareness of the tobacco quit helpline increased from 2129/3061 (69.6%) to 5844/6047 (96.6%), and knowledge of human papillomavirus (HPV) vaccination improved from 1889/3028 (62.4%) to 5744/6047 (95.0%).

Conclusion

Structured school-based interventions such as Project Udaan were associated with substantial improvement in adolescents’ awareness of tobacco-related health risks and supported early prevention strategies.

## Introduction

Tobacco consumption remains one of the leading preventable causes of disease and death in India, contributing substantially to the growing burden of cancer, lung disease, cardiovascular disease, and stroke [1]. Despite several national efforts, the initiation of tobacco use often occurs during adolescence [2], a formative period when habits are shaped by curiosity, peer pressure, and social influence. This early initiation substantially increases the lifetime risk of tobacco dependence and related diseases.

India accounts for nearly one-third of the world’s oral cancer burden [3], with tobacco use being the single most important risk factor [1]. The Global Youth Tobacco Survey (GYTS-4, 2019) revealed that one in eight tobacco users aged 20 to 34 years started smoking before 15 years of age [2]. These findings highlight adolescence as a critical window for preventive interventions aimed at reducing tobacco initiation and promoting long-term healthy behaviors.

Educational institutions, being the first organized social environment for young people, provide an effective platform for early intervention. Schools play a pivotal role in shaping health-related attitudes and behaviors, influencing both knowledge and social norms among students. In India, national frameworks such as the National Tobacco Control Programme (NTCP) and the Cigarettes and Other Tobacco Products Act (COTPA, 2003) mandate tobacco-free environments in educational institutions [4,5]. However, on-ground implementation of these policies often requires additional community engagement, structured awareness programmes, and active educator participation, gaps that targeted school-based interventions can effectively address.

Recognizing this need, Farmson Pharmaceutical Gujarat Pvt. Ltd., through its Corporate Social Responsibility (CSR) arm, the K. K. Vithani Foundation, initiated the School-based Oral Cancer Prevention Programme, implemented by Niramay Charitable Trust and technically supported by Fulcrum-Capitalizing CSR in collaboration with the District Education Department. The initiative aims to reduce tobacco use among students through awareness generation, capacity building of educators, and sustained behavior change interventions. The project was implemented across 100 schools in Vadodara district, Gujarat, India, targeting students across multiple grade levels.

The educational approach used in Project Udaan was informed by principles of behavior change communication and participatory learning theory. Interactive learning methods such as demonstrations, role plays, games, and peer discussions are widely recognized as effective strategies for improving knowledge retention and influencing health-related attitudes among adolescents. By combining cognitive learning with experiential engagement, the program aimed to enhance students’ awareness, reinforce risk perception, and encourage positive health behaviors related to tobacco prevention.

Against this background, the present study was undertaken to evaluate the effectiveness of Project Udaan, a structured school-based health education intervention. The primary objective of this study was to describe the implementation of Project Udaan and assess changes in students’ knowledge related to tobacco use and oral cancer prevention among students in Grades 6 to 10 using a pre-test and post-test design.

## Materials and methods

Study design and implementation of Project Udaan

Project Udaan was implemented as a structured school-based health education intervention across urban and rural schools in Vadodara district, Gujarat, India. The programme targeted students from Grades 6 to 10 and aimed to improve knowledge related to tobacco use, oral cancer prevention, and healthy behaviours through interactive and activity-based learning methods.

The study employed a pre-test and post-test cross-sectional design to assess changes in students’ knowledge following the intervention. The programme was implemented in two sequential phases between June 2025 and February 2026, with standardised educational activities conducted in participating schools.

Phase 1 Implementation

Phase 1 of Project Udaan focused on foundational awareness and structured technical education on tobacco use and oral cancer prevention. Each participating school received a standardised educational session consisting of multiple components delivered in sequence.

The session began with registration of participating students and teachers, followed by an introduction and ice-breaking activity designed to engage students and create an interactive learning environment. A pre-test questionnaire was then administered to assess baseline knowledge related to tobacco use and cancer prevention.

The core component of Phase 1 consisted of a technical presentation lasting approximately 20-25 minutes, covering topics including harmful effects of tobacco use, the association between tobacco and head and neck cancer, early warning signs of oral cancer, and awareness of human papillomavirus (HPV) vaccination and cervical cancer prevention.

Following the technical presentation, a live demonstration was conducted to illustrate the harmful effects of smoking using a visual lung simulation model involving a bottle and cigarette experiment to demonstrate smoke exposure and lung damage. Educational reinforcement was provided through a short awareness video titled “Bhailu’s Dream", which highlighted the long-term consequences of tobacco use on personal aspirations and health.

The session concluded with an interactive question-and-answer discussion, allowing students to clarify doubts and reinforce key messages. Upon completion of the session, participating schools issued completion certification, acknowledging programme implementation.

The total duration of Phase 1 sessions ranged from approximately 75 to 90 minutes, ensuring standardised delivery across schools.

These structured activities were designed to provide foundational knowledge, improve awareness, and promote early recognition of tobacco-related health risks among students. The components and duration of Phase 1 activities were standardised across schools to maintain uniformity of intervention delivery.

Phase 2 Implementation

Phase 2 of Project Udaan emphasised experiential learning, behavioural reinforcement, and participatory activities designed to strengthen students’ understanding of tobacco-related risks and encourage long-term behaviour change.

Each Phase 2 session began with student registration, followed by a series of structured interactive learning activities.

An interactive “Health Detectives” card-based game was conducted in which students identified potential health conditions such as stained teeth, mouth ulcers, or voice changes, linked them to possible risk behaviours including tobacco use, and discussed preventive strategies.

This was followed by an oral cancer screening role-play activity, where facilitators simulated doctor-patient interactions to demonstrate identification of early symptoms and appropriate preventive actions.

To illustrate behavioural aspects of addiction, a simulation exercise titled “Hooked!” was conducted, in which students participated in a structured clap-based activity triggered by auditory cues. This activity demonstrated the concept of habitual behaviour and the difficulty of stopping addictive actions.

Students also participated in the “Future 2035” time-capsule exercise, a guided visualisation activity designed to help students reflect on long-term consequences of tobacco use and imagine alternative healthy futures.

Additional participatory activities included the “Tobacco-Free Wall”, where students expressed personal reasons for remaining tobacco-free, and the “Count the Cost” exercise, which demonstrated the long-term financial burden associated with tobacco use by projecting cumulative spending over multiple years.

At the conclusion of Phase 2 activities, a post-test questionnaire was administered to assess knowledge following exposure to the intervention.

The total duration of Phase 2 sessions was approximately 75 to 90 minutes, with all activities delivered using standardised instructions and materials to ensure consistency across schools.

These structured experiential activities reinforced key health messages and strengthened behavioural awareness related to tobacco prevention among participating students.

School selection and participant recruitment

Participating schools were identified in coordination with the District Education Department, Vadodara. Schools included in the programme were those that consented to participate in the school-based cancer prevention initiative.

All students present in Grades 6 to 10 on the day of programme implementation were invited to participate in the educational session and knowledge assessment. Student participation in the questionnaire-based assessment was voluntary. No exclusion criteria were applied.

Pre-test responses were obtained from 6,017 students, and post-test responses were obtained from 6,047 students across participating schools.

Educator training and standardisation

All educators delivering the intervention underwent structured orientation and training prior to programme implementation. Training sessions included instruction on tobacco control concepts, adolescent health communication strategies, use of standardised presentation materials, and facilitation of interactive learning activities.

Educators were trained on standardised methods of facilitation and outlines of sessions to ensure uniform delivery of programme content across all schools. Regular coordination and supervision were conducted to maintain consistency in implementation across both phases of the programme.

Questionnaire design

A structured questionnaire was developed to assess students’ knowledge related to tobacco use and oral cancer prevention. The questionnaire consisted of multiple-choice items covering key domains, including: Identification of tobacco products; Association between tobacco use and oral cancer; Recognition of early symptoms of oral cancer; Understanding of peer pressure as a risk factor; Awareness of tobacco cessation helpline services; Knowledge of HPV vaccination as a preventive measure

The same questionnaire was used for both pre-test and post-test assessments to allow comparison of responses. The questionnaire is provided in Appendix A.

Timing of assessments

The pre-test questionnaire was administered immediately before the start of the educational session during Phase 1. The post-test questionnaire was administered immediately after completion of the educational session during Phase 2.

Questionnaires were completed anonymously without individual identifiers. Therefore, pre-test and post-test responses represent independent cross-sectional group-level responses rather than paired individual responses.

Participant flow and denominators 

Pre-test and post-test assessments were conducted among students present on the day of programme implementation. As participation was voluntary and questionnaires were completed anonymously, the number of responses varied across individual questionnaire items due to incomplete responses. Therefore, denominators differ slightly between knowledge domains and reflect the number of valid responses available for each item.

The reported numbers of schools and student participants represent those who attended the educational sessions during each implementation phase. Differences in participation counts between phases reflect variations in school schedules, student attendance, and programme timing.

Data collection and handling

Completed questionnaires were collected by trained facilitators following each session. Responses were compiled and entered into a structured Microsoft Excel database (Microsoft Corp., Redmond, WA, USA) for analysis.

Only completed responses for each individual question were included in percentage calculations. Incomplete or missing responses were excluded from analysis for the respective question but retained for other completed responses.

Data analysis

Data were analysed using descriptive statistical methods. Frequencies and percentages were calculated for each knowledge domain in both pre-test and post-test assessments.

Percentage-point differences between pre-test and post-test responses were calculated to assess changes in students’ knowledge following the intervention. Improvements in knowledge were described as substantial improvements based on observed percentage-point differences.

No inferential statistical tests were applied, as the analysis focused on descriptive comparison of group-level responses.

Ethical considerations

The activities described in this study were conducted as part of a school-based health education programme implemented with permission from participating schools and the District Education Department.

Student participation in the pre-test and post-test assessments was voluntary, and responses were collected anonymously to maintain confidentiality. No personal identifying information was collected at any stage of the study.

## Results

Project Udaan was implemented in two phases between June 2025 and February 2026. A total of 100 schools were covered during Phase 1 (June-October 2025) and 101 schools during Phase 2 (December 2025-February 2026) (Table 1). Rural schools constituted the majority in both phases, accounting for 63 schools in Phase 1 and 67 schools in Phase 2, while 37 urban schools participated in Phase 1 and 34 urban schools in Phase 2 (Table 1).

**Table 1:** Comparison of School Coverage and Student Participation Between Phase 1 and Phase 2 of Project Udaan

Variable	Category	Phase	Phase 2
Duration	—	June to October 2025	December 2025 to February 2026
Schools Covered	Urban	37	34
	Rural	63	67
	Total	100	101
Type of Schools	Primary	94	95
	Secondary	6	6
	Total	100	101
Children Covered	Urban	2186	1831
	Rural	4578	4354
	Total	6764	6185
Average Students per Session	—	67	61

Overall, 6764 children were covered during Phase 1 and 6185 children during Phase 2 (Table 1). Rural students represented the larger proportion in both phases, with 4578 rural students in Phase 1 and 4354 rural students in Phase 2, compared to 2186 urban students in Phase 1 and 1831 urban students in Phase 2 (Table 1). The average number of students per session was 67 in Phase 1 and 61 in Phase 2, indicating consistent participation across both implementation periods (Table 1). 

Variations in denominators across knowledge domains reflect differences in the number of completed responses for individual questionnaire items. Percentages were calculated based on valid responses available for each item.

Comparative analysis of pre-test and post-test responses demonstrated substantial improvement in students’ knowledge across multiple domains related to tobacco use and cancer prevention. Awareness about tobacco products increased from 5172/6017 (86.0%) in the pre-test to 5885/6047 (97.3%) in the post-test, representing an improvement of 11.4 percentage points (Table 2). Similarly, knowledge regarding the association between tobacco use and oral cancer improved from 5295/5963 (88.8%) to 5903/6047 (97.6%), showing an improvement of 8.8 percentage points (Table 2).

**Table 2:** Comparison of Pre-test (Phase 1) and Post-test (Phase 2) Knowledge on Tobacco Use and Cancer Prevention Among School Students HPV: human papillomavirus

Knowledge Domain	Pre-test n/N (%)	Post-test n/N (%)	Percentage Point Change
What is Tobacco?	5172/6017 (86.0%)	5885/6047 (97.3%)	11.4
Tobacco Causes Oral Cancer	5295/5963 (88.8%)	5903/6047 (97.6%)	8.8
Early Symptoms of Oral Cancer	4038/5983 (67.5%)	5643/6047 (93.3%)	25.8
Knowledge of Peer Pressure	2710/5926 (45.7%)	5377/6047 (88.9%)	43.2
Awareness of the Tobacco Quit Helpline	2129/3061 (69.6%)	5844/6047 (96.6%)	27.1
Awareness of HPV Vaccination	1889/3028 (62.4%)	5744/6047 (95.0%)	32.6

Recognition of early symptoms of oral cancer showed marked improvement, increasing from 4038/5983 (67.5%) in the pre-test to 5643/6047 (93.3%) in the post-test, reflecting an improvement of 25.8 percentage points (Table 2). Understanding of peer pressure as a factor influencing tobacco use demonstrated the greatest improvement, rising from 2710/5926 (45.7%) to 5377/6047 (88.9%), corresponding to an increase of 43.2 percentage points (Table 2).

Awareness of the government tobacco quit helpline improved from 2129/3061 (69.6%) to 5844/6047 (96.6%), showing an increase of 27.1 percentage points (Table 2). Additionally, awareness of HPV vaccination as a preventive measure against cervical cancer increased from 1889/3028 (62.4%) in the pre-test to 5744/6047 (95.0%) in the post-test, reflecting an improvement of 32.6 percentage points (Table 2).

Overall, these findings indicate a significant enhancement in students’ knowledge following the implementation of the structured school-based intervention. 

## Discussion

The findings from Project Udaan demonstrate that structured, school-based health education interventions can substantially improve adolescents’ knowledge related to tobacco prevention and cancer awareness. The substantial improvements observed in awareness of peer pressure, recognition of early symptoms of oral cancer, and understanding of tobacco cessation services suggest that participatory and activity-based learning approaches are effective in enhancing preventive health literacy among adolescents.

Strengths of the study

This study has several important strengths that enhance its relevance to public health practice. First, the intervention was implemented at a large scale across more than 100 schools, covering several thousand students from both urban and rural settings. The inclusion of diverse school environments improves the generalisability of findings within similar district-level contexts.

Second, the intervention utilised participatory and experiential learning methods, including demonstrations, role plays, games, and visual activities, which are well-suited for adolescent audiences and consistent with established health education approaches.

Third, the programme was closely aligned with national tobacco control policies, including the NTCP [4] and Tobacco-Free Educational Institution (TFEI) guidelines [6]. This alignment enhances the operational feasibility and scalability of the intervention within existing education and public health systems.

Finally, the structured multi-phase design allowed reinforcement of key health messages and provided an opportunity to deliver standardised educational content across multiple schools.

The marked improvement in understanding peer pressure observed in this study is particularly noteworthy. Adolescence represents a vulnerable period for experimentation with tobacco, largely influenced by peer dynamics, social acceptance, and behavioural modelling [7]. Previous studies have consistently reported that peer influence remains one of the strongest predictors of tobacco initiation among adolescents [8]. The improvement seen in this domain highlights the importance of incorporating behavioural and social learning strategies into school-based health programmes, rather than relying solely on informational approaches.

The high levels of improvement in recognition of early symptoms of oral cancer and awareness of tobacco-related health risks are consistent with findings from similar school-based interventions conducted in low- and middle-income countries. Educational interventions that use visual demonstrations, role-play, and interactive communication have been shown to substantially enhance retention of health information and encourage positive health behaviours among students [9].

Another important finding of this study is the substantial improvement in awareness of the government tobacco quit helpline and HPV vaccination as preventive strategies. Increasing awareness of cessation support services and preventive vaccination is essential in bridging the gap between knowledge and action [10]. This is particularly relevant in India, where utilisation of tobacco cessation services remains relatively low despite their availability.

The successful implementation of Project Udaan across both urban and rural schools demonstrates the feasibility of delivering standardised health education interventions across diverse settings. Rural participation accounted for the majority of students, indicating strong engagement in resource-limited settings where access to preventive health education may otherwise be limited. This highlights the scalability of the programme and its potential for expansion to additional districts and states.

Furthermore, the alignment of Project Udaan with national policy frameworks such as the NTCP [4], TFEI guidelines [6], and the COTPA [5] strengthens its relevance and sustainability. Integrating policy-driven frameworks into school-level implementation enhances institutional ownership and increases the likelihood of long-term programme continuation.

Despite the positive findings, sustained behavioural change requires repeated exposure to preventive messages and reinforcement through school policies and community engagement. Long-term follow-up studies are necessary to evaluate whether improved knowledge translates into reduced tobacco initiation and improved health behaviours over time.

The findings of this study demonstrate consistent improvements in knowledge across multiple tobacco and cancer prevention domains following implementation of the educational intervention. However, given the cross-sectional pre-test and post-test design and the absence of a control group, these findings should be interpreted as evidence of improved awareness associated with programme implementation rather than definitive proof of causal impact. While increased knowledge represents an essential first step in behaviour change pathways, further longitudinal research is required to determine whether these improvements translate into sustained behavioural outcomes and long-term reduction in tobacco use.

In addition to knowledge improvements, this study demonstrates the feasibility of implementing standardised school-based health education interventions across diverse urban and rural school settings. The successful delivery of programme components across more than 100 schools highlights the operational practicality of structured preventive health education models within existing educational systems. These findings support the potential scalability of similar school-based interventions within district-level public health programmes.

Limitations

This study has several limitations that should be considered when interpreting the findings. First, the study employed a pre-test and post-test cross-sectional design without inclusion of a control group. As a result, observed improvements in knowledge cannot be attributed solely to the intervention, and causal relationships cannot be firmly established.

Second, questionnaires were completed anonymously, and individual responses were not linked between pre-test and post-test assessments. Therefore, comparisons represent group-level changes rather than paired individual-level changes.

Third, the findings are based primarily on knowledge assessment rather than direct measurement of long-term behavioural changes among students. Although improved knowledge is an important first step, it does not necessarily translate into sustained behaviour change.

Fourth, the study was conducted across selected schools within a single district, which may limit generalisability to other geographic regions or educational contexts.

Fifth, differences in student participation between pre-test and post-test assessments resulted in varying denominators across questionnaire items. Only completed responses were included in calculations, which may influence comparability across measures.

Sixth, the analysis relied on descriptive statistical methods without the application of inferential statistical testing. Therefore, the observed improvements should be interpreted as descriptive changes rather than statistically confirmed differences.

Finally, the intervention consisted of multiple educational components delivered across different schools, making it difficult to determine which specific components contributed most strongly to observed knowledge improvements.

The findings of this study should be interpreted within the context of the study design. The absence of a control group and the use of independent cross-sectional pre-test and post-test responses limit the ability to establish causal relationships between the intervention and observed outcomes. Therefore, the observed improvements represent associations with programme implementation rather than definitive evidence of intervention effectiveness.

## Conclusions

Project Udaan demonstrates that structured school-based health education interventions can substantially improve adolescents’ knowledge related to tobacco use, cancer prevention, and healthy behaviours. The programme’s successful implementation across rural and urban schools highlights its feasibility and operational practicality within school-based health education systems.

The findings should be interpreted as evidence of improved knowledge and awareness following implementation rather than definitive proof of behavioural change or long-term health impact. Future studies incorporating control groups, paired longitudinal follow-up, and behavioural outcome measures are recommended to evaluate sustained programme impact.

## Figures and Tables

**Figure 1:**
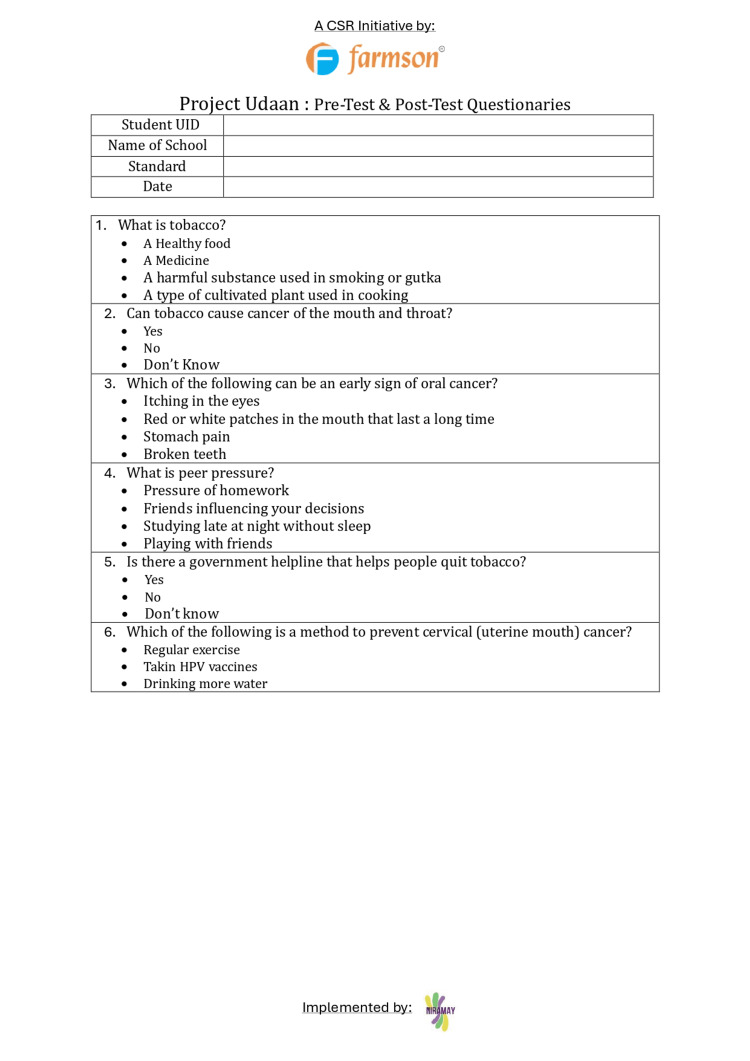
Project Udaan: Pre-test and Post-test Questionnaire

## Appendices

Appendix A 
